# Dicerandrol C Suppresses Proliferation and Induces Apoptosis of HepG2 and Hela Cancer Cells by Inhibiting Wnt/β-Catenin Signaling Pathway

**DOI:** 10.3390/md22060278

**Published:** 2024-06-14

**Authors:** Dongdong Zhou, Dandan Chen, Jingwan Wu, Ting Feng, Pinghuai Liu, Jing Xu

**Affiliations:** 1 Collaborative Innovation Center of Ecological Civilization, School of Chemistry and Chemical Engineering, Hainan University, Haikou 570228, China; zhoudongdonghf@163.com (D.Z.); 21220856000083@hainanu.edu.cn (D.C.); 20081700110009@hainan.edu.cn (J.W.); 20081700210004@hainanu.edu.cn (T.F.); twlph@163.com (P.L.); 2 Research and Utilization on Seaweed Biological Resources Key Laboratory of Haikou, Haikou 570228, China

**Keywords:** mangrove endophytic fungus, *Phomopsis asparagi*, dicerandrol C, apoptosis, Wnt/β-catenin signaling

## Abstract

Overwhelming evidence points to an aberrant Wnt/β-catenin signaling as a critical factor in hepatocellular carcinoma (HCC) and cervical cancer (CC) pathogenesis. Dicerandrol C (DD-9), a dimeric tetrahydroxanthenone isolated from the endophytic fungus *Phomopsis asparagi* DHS-48 obtained from mangrove plant *Rhizophora mangle* via chemical epigenetic manipulation of the culture, has demonstrated effective anti-tumor properties, with an obscure action mechanism. The objective of the current study was to explore the efficacy of DD-9 on HepG2 and HeLa cancer cells and its functional mechanism amid the Wnt/β catenin signaling cascade. Isolation of DD-9 was carried out using various column chromatographic methods, and its structure was elucidated with 1D NMR. The cytotoxicity of DD-9 on HepG2 and HeLa cells was observed with respect to the proliferation, clonality, migration, invasion, apoptosis, cell cycle, and Wnt/β-catenin signaling cascade. We found that DD-9 treatment significantly reduced tumor cell proliferation in dose- and time-dependent manners in HepG2 and HeLa cells. The subsequent experiments in vitro implied that DD-63 could significantly suppress the tumor clonality, metastases, and induced apoptosis, and that it arrested the cell cycle at the G_0_/G_1_ phase of HepG2 and HeLa cells. Dual luciferase assay, Western blot, and immunofluorescence assay showed that DD-9 could dose-dependently attenuate the Wnt/β-catenin signaling by inhibiting β-catenin transcriptional activity and abrogating β-catenin translocated to the nucleus; down-regulating the transcription level of β-catenin-stimulated Wnt target gene and the expression of related proteins including *p*-GSK3-β, β-catenin, LEF1, Axin1, *c*-Myc, and CyclinD1; and up-regulating GSK3-β expression, which indicates that DD-9 stabilized the β-catenin degradation complex, thereby inducing β-catenin degradation and inactivation of the Wnt/β-catenin pathway. The possible interaction between DD-9 and β-catenin and GSK3-β protein was further confirmed by molecular docking studies. Collectively, DD-9 may suppress proliferation and induce apoptosis of liver and cervical cancer cells, possibly at least in part via GSK3-β-mediated crosstalk with the Wnt/β-catenin signaling axis, providing insights into the mechanism for the potency of DD-9 on hepatocellular and cervical cancer.

## 1. Introduction

Cancer is a disease characterized by pathological and physiological changes in the inherent process of cell division, making it an imperative health concern. According to the Global Cancer Observatory (GLOBOCAN), it is estimated that 19.3 million people worldwide were diagnosed with cancer in 2020, resulting in 10 million deaths. Primary liver cancer, particularly hepatocellular carcinoma (HCC), is the sixth most common malignant tumor worldwide, accounting for approximately 4.7% of new cancer cases and 8.3% of all global deaths in 2020 [1]. Although systemic therapy, cytotoxin chemotherapy, immunotherapy, and oncolytic virus therapy have demonstrated survival benefits in the treatment of liver cancer, even for advanced HCC patients receiving the first-line target dug, sorafenib, the median overall survival is 11.5 months, and median progression-free survival is only 5.2 months with curative intent [2]. Cervical cancer (CC) is considered to be the fourth most frequently diagnosed cancer and the fourth leading cause of cancer death in women, with an estimated 604,000 new cases and 342,000 deaths in 2020 across the globe [1]. While vaccinations were introduced for the prevention of CC over the past several years, they provide limited benefits to eliminate preexisting infections or metastatic disease [3]. For patients treated with chemotherapy with or without bevacizumab, the survival time is still shorter (17 months), and associated with a rapid deterioration of quality of life due to toxicity profile [4]. Hence, it is not only of theoretical value but also of practical significance to search for candidate drugs having high efficiency and low toxicity along with mechanisms of action for overcoming HCC and cervical carcinoma.

The Wnt signaling pathway, broadly implicated in human cancers and experimental cancer models of animals, is a conserved signaling axis participating in diverse physiological processes such as proliferation, differentiation, apoptosis, metastases, and tissue homeostasis [5]. Wnt signaling pathways can be divided into three main branches: the canonical Wnt/β-catenin pathway, the non-canonical planar cell polarity (PCP) pathway, and the Wnt/Ca^2+^ pathway [6]. In the dysregulation of the canonical Wnt/β-catenin signaling pathway, the binding of Wnt ligands to Frizzled (Fz) receptors and the low-density lipoprotein-receptor-related protein 5/6 (LRP5/6) co-receptors induces the phosphorylation of Disheveled (Dvl) and prevents β-catenin destruction complex [composed of adenomatous polyposis coli (APC), Axin, glycogen synthase kinase-3β (GSK3-β), and casein kinase 1α (CK1α)]-dependent phosphorylation of β-catenin. Subsequently, stabilized β-catenin translocates into the nucleus and interacts with T-cell factor (TCF)/lymphocyte enhancer factor (LEF) [6]. The β-catenin/TCF/LEF transcription ultimately regulates the expression of Wnt/β-catenin target genes such as c-Myc and cyclin D1, thereby culminating in tumorigenic processes in the cells [7]. Evidence-based medicine has proposed the theoretical potential that efficient repression of this signaling might provide promising therapeutic choices in managing various types of cancers like liver, colorectal, cervical, breast, melanoma cancer, and so on [8]. Several small-molecule inhibitors and biologics for treating cancer have been developed for targeting of this pathway (e.g., natural agents: curcumin, 3,3 diindolylmethane, phytoestrogen; synthetic/small Wnt inhibitors: Rofecoxib; PRI-724, CWP232291; and monoclonal antibody against frizzled receptors, Vanituctumab) [9], but currently the only Wnt inhibitor in clinical testing is the LGK974 molecule (NCT01351103) [10].

Mangrove ecosystems are widely distributed in the intertidal zone of tropical and sub-tropical estuaries and coasts, containing abundant biological communities, for example, mangrove endophytic fungi, featuring various bioactive secondary metabolites with promising pharmaceutical applications [11]. Across the globe, coastal areas are primarily dominated by mangrove forests, which offer an intensely complex environment and biological potential of different endophytic fungal species that mostly remain unexplored [12]. Recently, a mangrove endophytic strain of *Phomopsis asparagi* DHS-48 isolated from the fresh root of *Rhizophora mangle* attracted our attention for the characterization of a series of immunosuppressive chromones [13], cytochalasins [14,15], and dimeric xanthones [16]. In order to tap the metabolic and biological potential of this titled fungal strain, epigenetic manipulation was used on this fungus to activate cryptic or silent genes by using histone deacetylase (HDAC) inhibitor sodium butyrate (at concentration of 50 μM). Our primary application of in vitro anti-tumor activity screening indicated that the EtOAc extracts were significantly enhanced after sodium butyrate regulation towards human hepatocellular and cervical cancer cell lines HepG2 and HeLa (IC_50_ values of 10.45 ± 0.17 μg/mL and 11.25 ± 0.58 μg/mL, respectively) at the concentration of 10 μg/mL. Bioassay-guided investigation of the anti-tumor constituents obtained from the large-scale fermentation of the abovementioned manipulated DHS-48 resulted in the isolation of dicerandrol C (DD-9). In this study, we aimed to explore the underlying mechanisms through which DD-63 regulates the Wnt/β-catenin signaling cascade, causing decreased proliferation, clonality, migration, invasion, and induced apoptosis of hepatocellular and cervical carcinoma cell lines.

## 2. Results

### 2.1. Identification of DD-9

**Figure 1:**
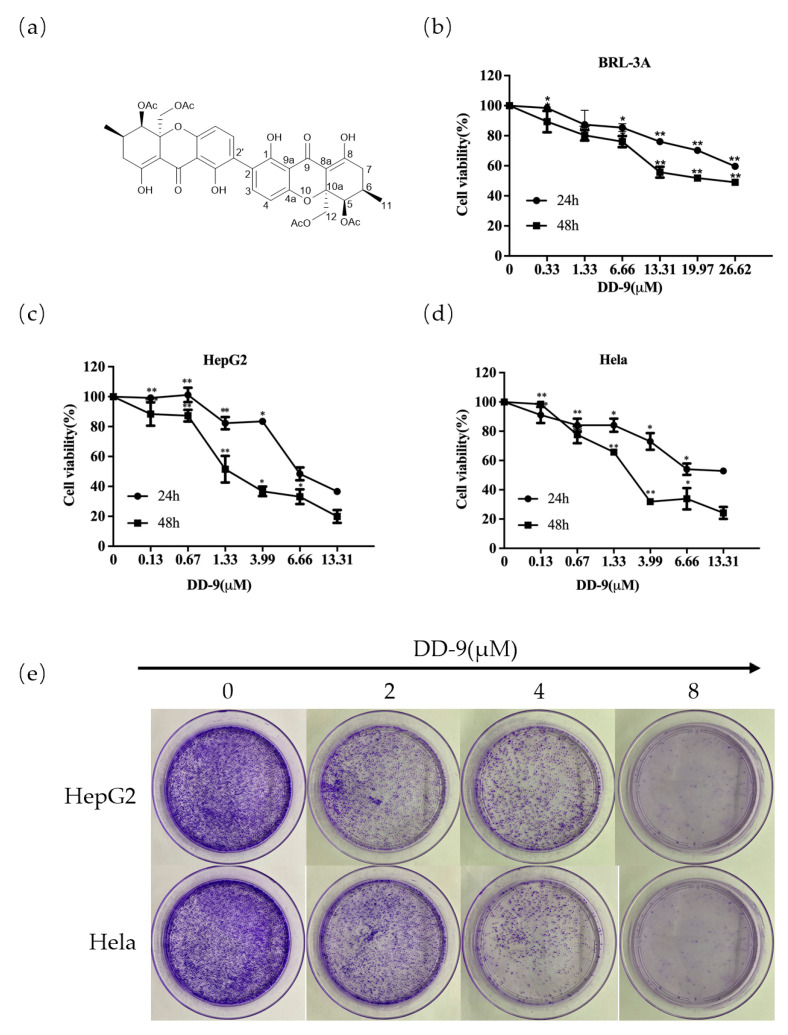
Effects of DD-9 on the growth of cells. (**a**) Chemical structure of DD-9. (**b**) Cytotoxic effects of DD-9 on normal rat liver cell. (**c**) Cytotoxic effect of DD-9 on HepG2 cells. (**d**) Cytotoxic effect of DD-9 on HeLa cells. (**e**) Effect of DD-9 on clone formation. Data are presented as the mean ± SD of three independent tests. * *p* < 0.05, ** *p* < 0.01 compared with the control group.

Dicerandrol C (DD-9): light yellow amorphous powder (MeOH); [α]^20^_D_ -90 (c 0.0001, MeOH); UV (MeOH) λ_max_ 205,206,207 nm; ^1^H NMR (400 MHz, CDCl_3_) δ_H_ 14.04 (2H, s, 8-OH, 8′-OH), 11.80 (2H, s, 1-OH, 1′-OH), 7.39 (2H, d, J = 8.5 Hz, H-3, H-3′), 6.46 (2H, d, J = 8.5 Hz, H-4, H-4′), 5.56 (2H, s, H-5, H-5′), 4.49 (2H, d, J = 12.8 Hz, Ha-12, Ha-12′), 4.18 (2H, d, J = 13.1 Hz, Hb-12, Hb-12′), 2.40–2.49 (4H, m, H_2_-7, H_2_-7′), 2.40 (2H, m, H-6, H-6′), 2.09 (12H, s, H_3_-14, H_3_-14′, H_3_-16, H_3_-16′), 1.06 (6H, d, J = 5.9, H_3_-11, H_3_-11′); ^13^CNMR (100 MHz, CDCl_3_) δ_C_ 187.8 (C-9, C-9′), 177.7 (C-8, C-8′), 170.4 (C-13, C-13′), 170.4 (C-15, C-15′), 159.3 (C-1, C-1′), 157.4 (C-4a, C-4a′), 140.2 (C-3, C-3′), 117.8 (C-2, C-2′), 108.1 (C-4, C-4′), 106.3 (C-9a, C-9a′), 100.4 (C-8a, C-8a′), 80.5 (C-10a, C-10a′), 70.4 (C-5, C-5′), 65.3 (C-12, C-12′), 33.4 (C-7, C-7′), 27.7 (C-6, C-6′), 20.8 (C-14, C-14′), 20.7 (C-16, C-16′), 17.6 (C-11, C-11′). HRESIMS *m*/*z* [M + H]^+^ 751.2235 (calcd for C_38_H_37_O_16_ 751.2238). The chemical structure of DD-9 is shown in Figure 1a.

### 2.2. DD-9 Inhibited the Viability and Proliferation of HepG2 and HeLa Cells In Vitro

HepG2 and HeLa cells as well as rat normal liver BRL-3A cells were exposed to increasing concentrations of DD-9 for different time periods (24 or 48 h). As demonstrated in Figure 1b, DD-9 treatment had minimal effects on the viability of normal liver BRL-3A cells after 24 and 48 h cultivation (IC_50_ values of 56.79 ± 0.22 μM and 26.40 ± 0.42 μM, respectively), but significantly reduced tumor cell proliferation in a concentration-dependent manner in both tested cancer cells (Figure 1c,d), indicating that DD-9 has a relatively low toxicity toward the survival of normal liver cells. Meanwhile, we compared the IC_50_ values of DD-9 in these two cell lines at different times (24 or 48 h). Our results manifested that DD-9 also inhibited the cancer cell viability time-dependently (IC_50_ values of 13.38 ± 1.13 μM and 4.17 ± 0.49 μM in HepG2 cells; IC_50_ values of 23.40 ± 6.98 μM and 5.18 ± 0.56 μM in HeLa cells) at 24 and 48 h. We further evaluated the long-term inhibitory effect of DD-9 on these two kinds of cells by colony formation assays, measured after treatment with respective concentrations of DD-9 for 48 h. These assays showed that DD-9 significantly concentration-dependently reduced the clone forming ability compared with the control group (Figure 1e).

### 2.3. DD-9 Inhibited the Migration and Invasion of HepG2 and HeLa Cells

**Figure 2:**
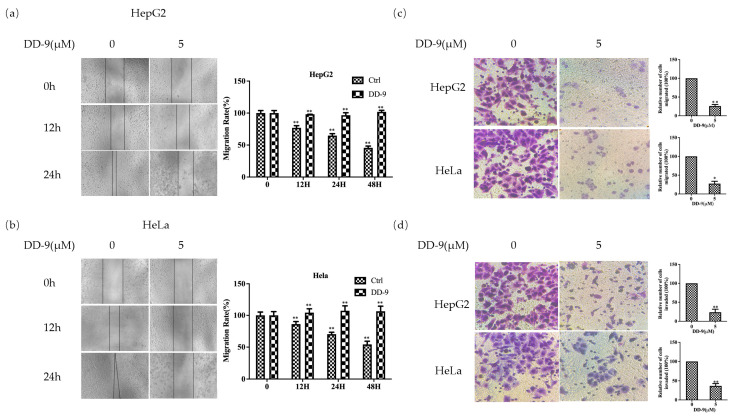
DD-9 inhibited the migration and invasion of HepG2 and HeLa cells. DD-9 inhibited the migration of HepG2 (**a**) and HeLa (**b**) cells. The cell monolayers were scraped off and incubated with 5 μM DD-9 for 12, 24, and 48 h before being photographed. After 24 h of DD-9 treatment, transwell assay was performed and the cells were observed under microscope at 200 times. HepG2 and HeLa cells were detected for migration (**c**) and invasion (**d**) and compared with the control group, * *p* < 0.05, ** *p* < 0.01.

To detect the effect of DD-9 on the cell metastatic capacity, cell scratch test, transwell migration, and invasion assays were performed. As shown in Figure 2a,b, a dose of DD-9 (5 μM) treatment effectively inhibited wound healing rate of HepG2 and HeLa cells when compared with their respective control groups at 12 and 24 h in a time-dependent manner. To further investigate the effect of DD-9 on HepG2 and HeLa cell migration, we performed transwell assay and our results showed that the number of migrated cells in the DD-9-treated HepG2 and HeLa cells was significantly decreased (Figure 2c,d). Similarly, HepG2 and HeLa cancer cell invasion through a Matrigel-coated membrane was likewise significantly impaired in response to DD-9 exposure. These results indicated that DD-9 inhibited the migration and invasion of HepG2 and HeLa cells in vitro.

### 2.4. DD-9 Induced Apoptosis of HepG2 and HeLa Cancer Cells

**Figure 3:**
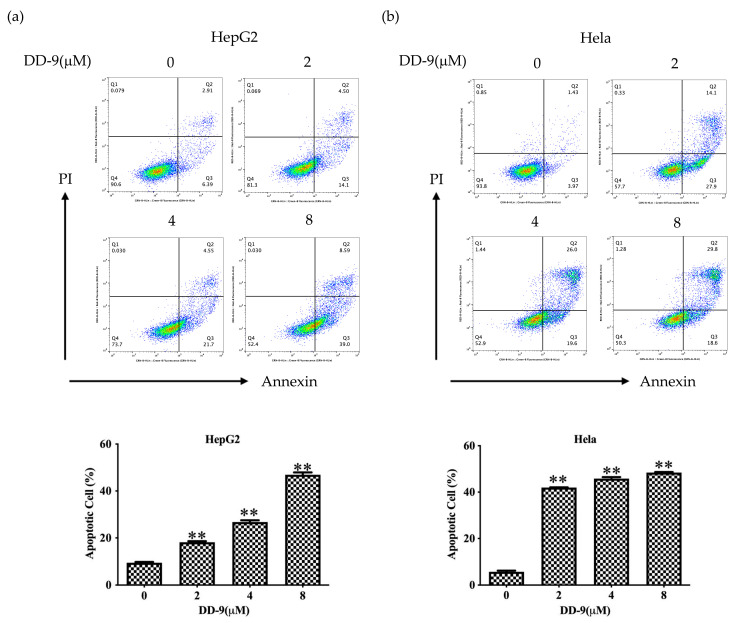
DD-9 induced HepG2 and HeLa cell apoptosis. HepG2 (**a**) and HeLa (**b**) cells were treated with different concentrations of DD-9 for 48 h, stained with Annexin V-FITC and PI for 30 min, and analyzed by flow cytometry. Annexin V positive populations are considered as cells undergoing apoptosis. Data are presented as the mean ± SD of three independent experiments. ** *p* < 0.01 compared with the control group.

The induction of apoptosis (programmed cell death) is a hallmark of the cellular response of many cancer cells to treatment with anticancer drugs. To determine whether the growth inhibitory activity of DD-9 was related to the induction of apoptosis, Annexin-V/PI double staining was performed using flow cytometry. The results showed that increased concentration of DD-9 treatment for 48 h considerably elevated the proportion of apoptotic cells (early as well as late apoptotic cells) (apoptosis rates of HepG2 9.50 ± 0.26%, 18.20 ± 0.44%, 26.76 ± 0.76%, and 46.93 ± 0.99%; HeLa apoptosis rates 5.72 ± 0.53%, 42 ± 0.10%, 45.83 ± 0.68%, and 48.43 ± 0.25%) for 0, 2, 4, and 8 μM, accordingly, as depicted in Figure 3a,b. The underlined results suggest that DD-9 induced apoptosis in HepG2 and HeLa cells in a dose-dependent manner.

### 2.5. DD-9 Stimulated G_0_/G_1_ Cell Cycle Arrest in HepG2 and HeLa Cells

**Figure 4:**
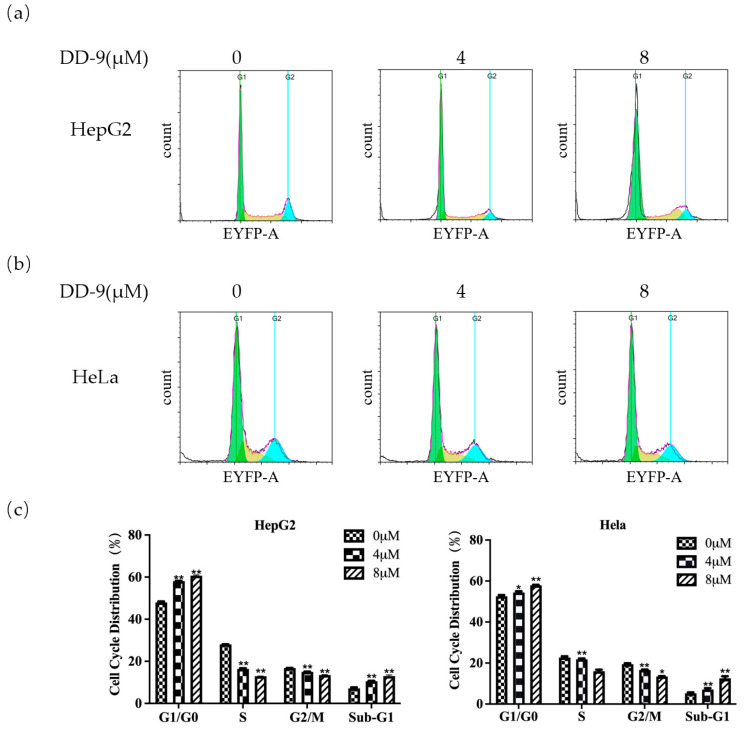
DD-9 stimulated cell cycle arrest in HepG2 and HeLa cells. HepG2 (**a**) and HeLa (**b**) cells were treated with 0, 4, and 8 μM DD-9 for 48 h, and cell cycles were determined by flow cytometry. (**c**) A statistical investigation was performed for changes of HepG2 and HeLa cell cycle at 48 h after DD-9 treatment. Data are presented as the mean ± SD of three independent experiments. * *p* < 0.05, ** *p* < 0.01 compared with the control group.

In addition to apoptosis, we also tested whether DD-9 could induce cell cycle arrest in HepG2 and HeLa cells by flow cytometry. Cancer cells treated with a serial concentration of DD-9 for 48 h were subjected to DNA pattern analysis. Results showed that the percentages of cells at G_0_/G_1_ phase at 48 h were in a dose-dependent manner 48.3 ± 0.44, 58.20 ± 0.16, and 60.25 ± 0.35% (in HepG2 cells) and 52.14 ± 0.56, 54.63 ± 0.61, and 58.19 ± 0.24% (in HeLa cells) at 0, 4, and 8 μM concentrations of DD-9, respectively (Figure 4a,b). Moreover, a dramatic increase of cell population in sub-G_0_ fraction was induced by 8 μM DD-9 treatment, suggesting that the anti-proliferative activity of DD-9 also resulted from apoptosis induction, while a considerable downward trend was observed in these cells in S-phase, indicating that the proliferation of HepG2 and HeLa cells were effectively inhibited by DD-9 through cell cycle arrest at the G_0_/G_1_ phase (Figure 4c).

### 2.6. DD-9 Inhibited the Activation Level of β-Catenin

**Figure 5:**
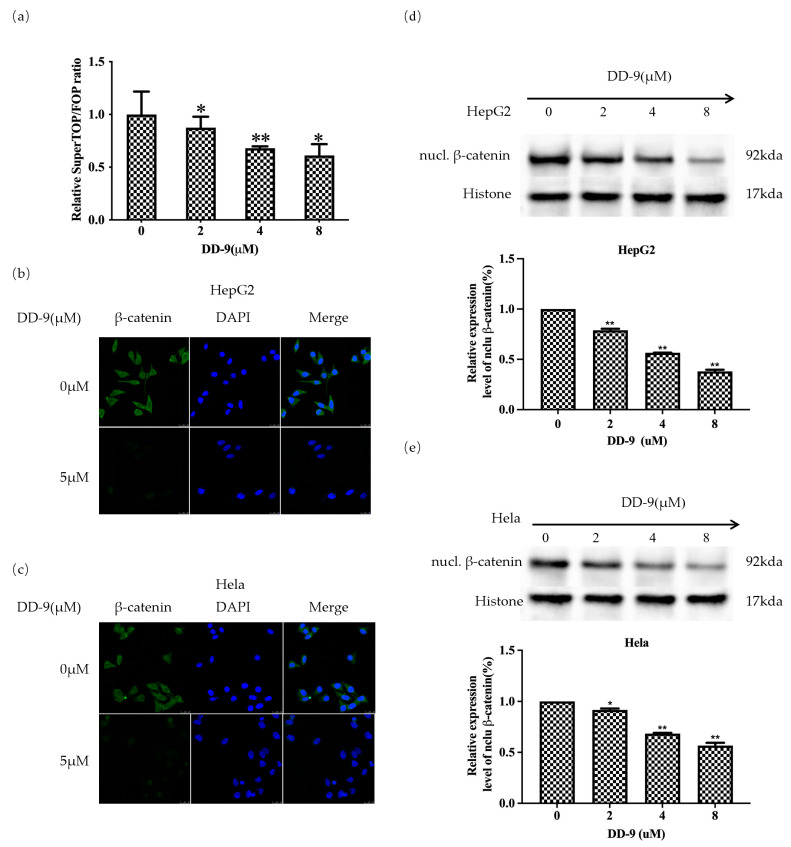
Identification of DD-9 as an inhibitor of the Wnt/β-catenin pathway. (**a**) Inhibitory effect of DD-9 on TOPflash/FOPflash activity in HEK293 cells. After treatment of HepG2 (**b**) and HeLa (**c**) cells with 5 μM DD-9 for 48 h, the levels of β-catenin were detected by immunocytochemical staining with FITC goat anti-rabbit IgG. Effect of DD-9 on nuclear β-catenin protein levels in HepG2 (**d**) and HeLa (**e**) cells. Results indicate the mean ± SD (n = 3) and represent data from three independent experiments. * *p* < 0.05, ** *p* < 0.01 compared with control group.

**Figure 6:**
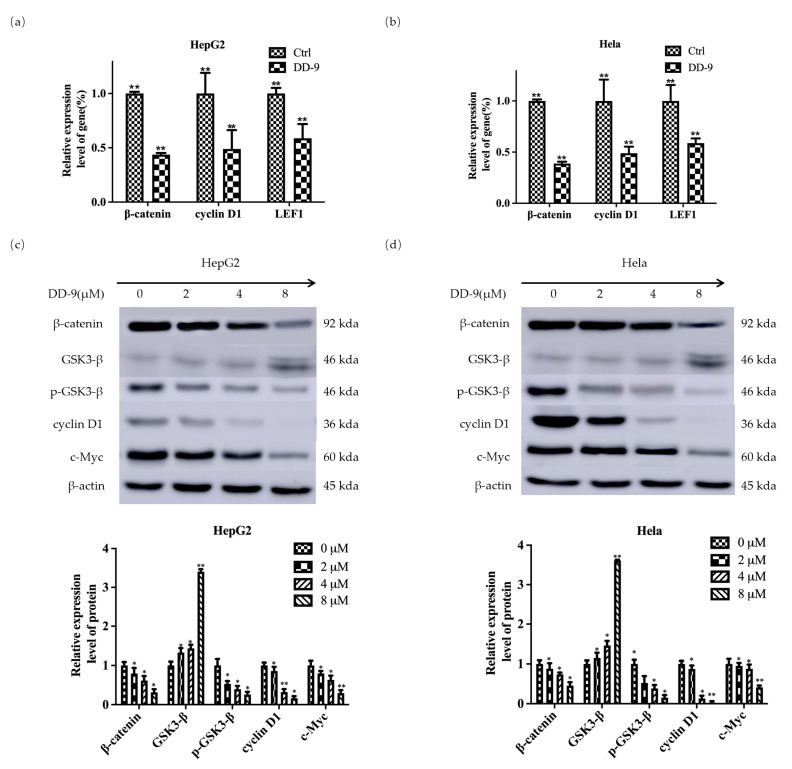
DD-9 suppressed the Wnt/β-catenin signaling cascade. The mRNA expression of β-catenin, cyclin D1, and LEF1 after 5 μM DD-9 treatment for 48 h in HepG2 (**a**) and HeLa (**b**) cells was detected by real-time quantitative PCR. Whole HepG2 (**c**) or HeLa (**d**) cell lysates were prepared and the levels of β-catenin, p-GSK3-β, GSK3-β, cyclinD1, and c-Myc were determined by Western blot analysis and normalized to β-actin. Results indicate the mean ± SD (n = 3) and represent data from three independent experiments. * *p* < 0.05, ** *p* < 0.01 compared with control group.

To uncover the underlying mechanism of DD-9 in HCC and cervical cancer, and whether it was related to the Wnt/β-catenin signaling pathway, we performed TOP/FOP-Flash luciferase reporter assay. DD-9 (Figure 5a) exhibited potent inhibitory activity against the TOPflash reporter gene in a concentration-dependent manner. In contrast, activity of FOP Flash, a negative control reporter with mutated β-catenin/TCF binding sites, was unchanged by treatment with DD-9, suggesting that DD-9 was a small molecule inhibitor of the Wnt/β-catenin signaling pathway and dose-dependently inhibited the activation level of the β-catenin. To further confirm the inhibitory effect of DD-9 on the Wnt/β-catenin signaling pathway, we conducted immunofluorescence assay to explore the distribution of β-catenin in HepG2 and HeLa cells. As shown in Figure 5b,c, the intensity of green fluorescence in the nuclei of both cancer cells was high in the control group. However, in the DD-9-treated groups, the intensity of green fluorescence in the cytoplasm and the nucleus was significantly decreased compared with the control group, indicating that DD-9 could inhibit the accumulation of β-catenin in the cytoplasm and attenuated its translocation from cytoplasm to the nucleus. Moreover, Western blot analysis was also conducted to detect the nuclear expression of β-catenin in cancer cells. It was clear that the accumulation of β-catenin in the nucleus was inhibited in a dose-dependent manner by the treatment of HepG2 and HeLa cells with DD-9 (Figure 5d,e), which further confirmed the inhibiting effect of DD-9 on nuclear translocation of β-catenin. Meanwhile, DD-9 can effectively reduce the mRNA transcription level of β-catenin (Figure 6a,b), indicating that DD-9 may inhibit Wnt signaling by directly interfering with β-catenin mRNA transcription in the nucleus.

### 2.7. DD-9 Suppressed Wnt/β-Catenin Signaling Cascade in HepG2 and HeLa Cells

To further demonstrate the molecular mechanism of DD-9 on the Wnt/β-catenin signaling pathway in HCC and CC, we investigated the effect of DD-9 by detecting expression of Wnt target genes and associated proteins in both HepG2 and HeLa cells. The results revealed that DD-9 can effectively reduce the mRNA expression of β-catenin and its target genes cyclin D1 and LEF1 and inhibit their transcription level, compared to that in the control group in tested cancer cells (Figure 6a,b). In addition, Western blot results showed that DD-9 treatment downregulated the protein expression levels of β-catenin, p-GSK3-β, cyclinD1, and c-Myc, and showed a marked increase in GSK3-β expression in a dose-dependent manner (Figure 6c,d). These data suggest the contribution of DD-9 against the proliferation of liver and cervical cancer cells mediated through GSK3-β activation, and enhancement of protein degradation, i.e., β-catenin, LEF1, p-GSK3-β, cyclinD1, and c-Myc of Wnt/β-catenin signaling cascade, as well as reduction of the nuclear accumulation of β-catenin.

### 2.8. Molecular Docking Analysis of the Binding Interaction of DD-9 with β-catenin and GSK3-β

**Figure 7:**
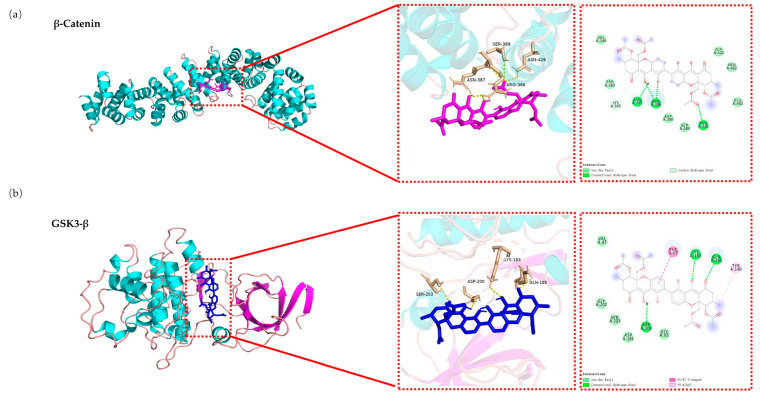
The molecular docking analysis of DD-9 binding to β-catenin (**a**) and GSK3-β (**b**) were performed using the AutoDock 4.2 program, simulating their interaction at a molecular level; green dotted lines, H-bonds labeled with distances in Å; purple line, pi-hydrophobic interaction.

We next performed molecular docking studies to determine the orientation of DD-9 bound in the active sites of β-catenin and GSK3-β, using the AutoDock 4.2 program as described. The analysis revealed that DD-9 was docked within the β-catenin protein and the binding energy was −9.7 kcal/mol, and as shown in Figure 7a, it formed three hydrogen bonds with ASN387, ARG386, and ASN426 residues of β-catenin. Additionally, the compound demonstrated van der Waals interactions with the following residues of β-catenin: Val349, Trp383, Ser389, Asp390, Gly422, Pro463, and Glu462. Meanwhile, DD-9 showed greater binding affinity with GSK3-β when compared to β-catenin, with a considerably high binding energy of −11.3 kcal/mol. Figure 7b depicts the interaction between DD-9 and GSK3-β, where it formed three hydrogen bonds with Asp200, Lys183, and Gln185 residues. The Phe67 residue of GSK3-β participated in a T-shaped π–π interaction to enhance binding affinity, while Tyr140 was involved in a π–alkyl interaction for the same purpose. Moreover, DD-9 exhibited van der Waals forces with the following residues of GSK3-β: Val87, Gly202, Gly65, Ser203, and Asp181. Overall, these findings further confirm that DD-9 exerted its antitumor effects via GSK3-β-mediated crosstalk with the Wnt/β-catenin signaling pathway.

## 3. Discussion

Metabolites from mangrove endophytic fungi encode unique biosynthetic genes which have hogged the limelight in drug discovery because of their promise as therapeutic agents [17]. In recent years, many structurally diverse compounds with unique skeletons have been identified from mangrove endophytic fungi and evaluated for their antiproliferative properties [12]. Some of them have as of now been acknowledged as potential anticancer drug lead candidates, such as paeciloxocin A [18], sumalarins A–C [19], merulin A and C [20], bostrycin [21], 1403P-3 [22], SZ-685C [23], deoxybostry [18], brocazine G [18], and Sdy-1 [24,25]. Although these compounds may serve as lead molecules for the development of new anticancer drugs, none of them has reached the market yet. These facts inspire both pharmaceutical researchers and natural product chemists to identify new anticancer agents from mangrove endophytic fungi.

Dicerandrol C (DD-9) was first reported from the culture broth of the *Phomopsis longicolla*, an endophytic fungus from the mint species *Dicerandra frutescens* [26], and was later isolated from different cultures of *Phomopsis* sp. [27,28,29]. As a symmetric tetrahydroxanthenone dimer, DD-9 has been shown to exhibit significant antimicrobial activity against *Staphylococcus aureus* (ATCC 6538) and *Staphylococcus saprophyticus* (ATCC 15305) [29], but weak activity against *Bacillus subtilis* [26]. This compound also displays cytotoxic activity against human cancer cell lines MDA-MB-435, HCT-116, Huh7, A549, and MCF-10A (breast, colon, liver, lung, and mammary epithelial tumor, respectively) [26,27] and murine lymphoma cell L5178Y, which is slightly pro-apoptotic to human lymphoma-derived DG75b and Jurkat T cells [30]. However, the action mechanisms of DD-9 remain to be clarified. In this study, primary anti-tumor activity screening indicated that chemical epigenetic manipulation of the endophytic fungus *Phomopsis asparagi* DHS-48 obtained from mangrove plant *Rhizophora mangle* enhanced the cytotoxicity of its EtOAc extracts towards human hepatocellular and cervical cancer cell lines HepG2 and HeLa. Bioassay-guided investigation of titled fungal strain DHS-48 led to the isolation of DD-9. Its structure was unequivocally determined by 1D NMR spectroscopic experiments as well as mass spectrometry (Figures S1–S4) and comparison with data reported in the literature [26].

Previous studies demonstrated that inhibition of tumor cell proliferation and colony formation play a major role in suppression of malignancies [31]. Accordingly, DD-9 was herein confirmed to hamper HepG2 and HeLa cell proliferation, supporting its utility as a potent anti-cancer drug candidate, having caused more significant cytotoxic cell death for hepatocellular and cervical cancer cell lines as compared to control normal mouse hepatocytes. Its ability to suppress colony formation was further manifested. Cancer metastasis is the major cause of cancer-associated death [32]. Next, we demonstrated that DD-9 retarded cell migration and invasion of HepG2 and HeLa cells, which are generally seen as prerequisite events for cancerous metastasis [33]. Apoptosis and proliferation are intimately coupled, and deficient apoptosis is known to cause uncontrolled cell proliferation [34]. For over three decades, a mainstay and goal of clinical oncology has been the development of therapies promoting the effective elimination of cancer cells by apoptosis [35]. Flow cytometry evaluation of Annexin-V/PI binding during DD-9 treatment revealed that the percentages of (Annexin-V^−^/PI^−^) live cells, (Annexin-V^+^/PI^−^) early apoptotic cells, (Annexin-V^+^/PI^+^) late apoptotic cells, and (Annexin-V^−^/PI^+^) necrotic cells were in agreement with the previous report that DD-9 promotes apoptosis in DG75b cells and Jurkat T cells [30]. Moreover, after the cancer cells were stained with Annexin V labeled fluorescent dye FITC and PI to visualize the apoptotic events, significant apoptotic structural changes of nuclear fragmentation and chromatin granule condensation, which are the prominent characteristic features for apoptosis events, were observed. Therefore, all of the above experimental data clearly indicated that DD-9 significantly increased the apoptosis of HepG2 and HeLa cells. Based on the changes in cell DNA content, the growth and reproduction of proliferating cells can be well-defined into four stages, in which it grows (G1), duplicates its DNA (S phase), prepares to divide (G2), and divides (M phase) [36]. In our study, DD-9-treated HepG2 and HeLa cells showed the accumulation of a sub-diploid DNA peak in cell cycle distribution at the G_0_/G_1_ phase simultaneously with a reduction in the progression of S and G_2_/M phase, thereby blocking progression through the G_1_/S transition of the cell cycle.

**Figure 8:**
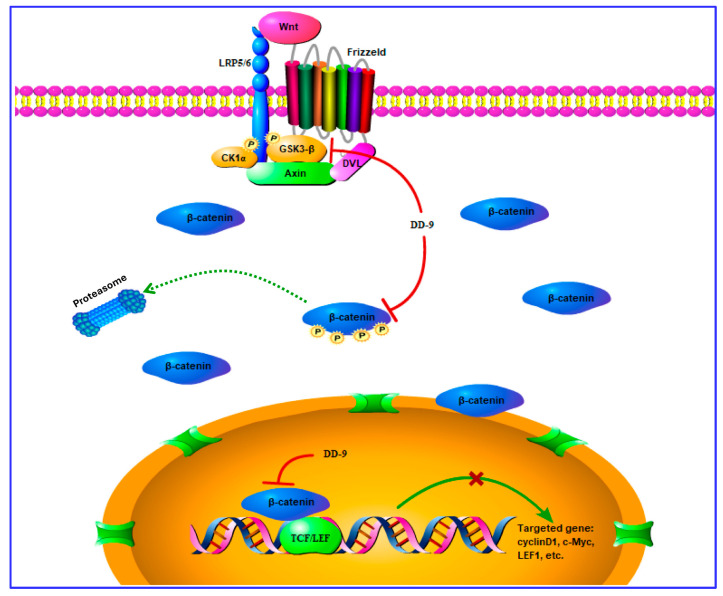
Potential demonstration of DD-9 inhibiting the activation of the Wnt/β-catenin signaling pathway.

Abnormal Wnt/β-catenin signaling is associated with various cancers and is considered an attractive mechanism-based antitumor therapeutic approach [37]. Oncogenic activation of Wnt signaling can ensue from a variety of distinct aberrations in the signaling pathway, but most share the common feature of causing increased cellular levels of β-catenin by interfering with its constitutive degradation [38]. Liver cancer is highly heterogeneous and β-catenin accumulation has been observed in about 10–50% in HCC, and has been correlated with tumor progression and poor prognosis [39]. Some studies have shown the involvement of certain Wnt/β-catenin signaling genes in CC pathogenesis. Among these Wnt signaling components, the imbalance in the structural and signaling properties of β-catenin are extensively characterized in CC [40]. TOPFlash reporter plasmid has been used to determine the β-catenin-mediated TCF/LEF transcription activity in the Wnt signaling cascade and has been widely used to study the Wnt signaling cascade [41]. Therefore, we performed TOP/FOPFlash luciferase reporter assay, and the results showed that DD-9 significantly decreased TOPFlash activity, implying that DD-9 can constrain the triggering of the Wnt/β-catenin cascade. Next, we conducted immunofluorescence assay to further explore the distribution of β-catenin in the HepG2 and HeLa cells after treatment with DD-9. The data demonstrated that DD-9 could inhibit the accumulation of β-catenin in the cytoplasm as well as its transfer to the nucleus. All the results indicated that DD-9 could inhibit the activation of the Wnt/β-catenin pathway. The abnormal excitation of the pathway involved accumulation of β-catenin in the cytoplasm affected by the reduced stability of the upstream GSK3-β-related β-catenin destruction complex, and then stimulation of the T cell factor/lymphocyte enhancer factor (TCF/LEF) to regulate Wnt responsive downstream target oncogene expression, including Axin1, *c*-Myc, and CyclinD1, which can lead to cell proliferation, inhibition of cell apoptosis, and tumor formation [37]. Accumulated evidence has confirmed that active GSK3-β was abruptly expressed in normal tissues but showed decreased expression in precancerous and cancerous lesions [42]. Cyclin D1 is a critical cell-cycle regulator, which was synthesized early in the G_1_ phase and is a target molecule transcriptionally activated by aberrant β-catenin in the Wnt signaling pathway [43]. c-Myc is also a cell-cycle regulator that promotes reprogramming of cancer cell metabolism in the G_1_ phase and also plays a key role in regulating apoptosis [44]. In this study, DD-9 has been shown as a potential candidate against hepatocellular and cervical cancer by downregulating the transcription level of β-catenin-stimulated Wnt target gene and the expression of related proteins including p-GSK3-β, β-catenin, LEF1, c-Myc, and CyclinD1; and by upregulating GSK3-β expression, which indicates that DD-9 stabilized the β-catenin degradation complex, thereby inducing β-catenin degradation and inactivation of the Wnt/β-catenin pathway. Consistent with our observation, molecular docking studies revealed that DD-9 exhibits favorable binding affinities towards the target proteins β-catenin and GSK3-β, displaying low energy values of −9.7 kcal/mol and −11.3 kcal/mol, respectively. Research has shown that β-catenin residues His260, Asn261, Lys292, Ile296, Asp299, Tyr306, Gly307, Lys312, Lys335, Lys345, Arg376, Arg386, Asn387, Asn426, Cys429, Lys435, Cys466, His470, Arg474, and Lys508 are the residues that interact with TCF4 to form a complex [45,46].Our molecular docking results indicate that DD-9 occupied an important binding site of β-catenin via the formation of hydrogen bonds with Arg386 and Asn426, which is a key TCF4/β-catenin interaction. In addition, the tetrahydroxanthenone ring of DD-9 also undergoes hydrophobic interactions with β-catenin. LEF1 is a transcription factor that promotes the canonical Wnt/β-catenin signaling pathway by cooperating with TCF4 [47]. Consequently, it is predicted that the ectopic expression of LEF1 was inhibited by DD-9 through suppression of β-catenin/TCF4 transcriptional activity. Meanwhile, it was known that GSK-3 inhibitors are of diverse chemotypes and mechanisms of action and include ATP-competitive inhibitors, non-ATP-competitive inhibitors, and substrate-competitive inhibitors [48]. Crystallographic data identified as Lys85, Asp133, Val135, Thr138, Lys183, Gln185, and Asp200 were critical residues in the ATP-binding site of GSK-3 [49]. A combined approach of mutagenesis and computational protein–protein docking analyses identified a novel substrate-binding site within the catalytic core of GSK3-β formed by Phe67, Gln89, Phe93, and Asn95, which were spatially located near the ATP binding site and the phosphate binding pocket that interacts with the phospho-serine moiety of the substrate [50,51]. Agreeing with these reports, our molecular docking study showed that DD-9 specifically interacted with Lys-183, Gln-185, and Asp-200 within or nearby the ATP-binding pocket GSK3-β. Phe67 is not directly involved in ATP binding [50], but it may be important for stabilizing the conformational change by ATP binding. Our finding indicated that DD-9 might be a kind of ATP competitive inhibitor of GSK3-β. Hence, our in silico results strongly coincide with the experimental results that exhibited its antitumor effects via GSK3-β-mediated crosstalk with the Wnt/β-catenin signaling cascade (Figure 8).

## 4. Materials and Methods

### 4.1. General Experimental Procedure

Optical rotation was recorded at 20 °C using an ATR-W2 HHW5 digital Abbe refractometer (Shanghai Physico-optical Instrument Factory, Shanghai, China). The UV spectra were obtained using a Shimadzu UV-2600 PC spectrophotometer (Shimadzu Corporation, Tokyo, Japan). ^1^H NMR and ^13^C NMR spectra were recorded on a Bruker AV-400 (Bruker Corporation, Fällanden, Switzerland) instrument with TMS as an internal standard. High-resolution ESI–MS were recorded on an Agilent 6210 mass spectrometer (Agilent Technologies, Waldbronn, Germany) employing peak matching. Silica gel (200–300 mesh, Qingdao Marine Chemical Factory, Qingdao, China) was used for column chromatography (CC). Thin-layer chromatography (TLC) was performed on precoated silica gel GF_254_ plates (Qingdao Marine Chemical Factory, Qingdao, China). Minimum Eagle’s Medium (MEM) and 3-(4,5-dimethylthiazol-2-yl)-2,5-diphenyltetrazolium bromide (MTT) were purchased from Thermo Fisher Scientific (Milwaukee, WI, USA). Fetal bovine serum (FBS) was bought from Hangzhou Sijiqing Biological Engineering Materials Co., Ltd. (Hangzhou, China). Radio Immunoprecipitation Assay (RIPA) lysis buffer, phenylmethylsulfonyl fluoride (PMSF), phosphate-buffered saline (PBS), and parenzyme were supplied by Boster Biological Technology Co., Ltd. (Wuhan, China). Primary antibodies against β-catenin, phosphor-GSK3-β, GSK3-β, Cyclin D1, and c-Myc and anti-rabbit IgG and HRP-linked antibody were purchased from Cell Signaling Technologies (Danvers, MA, USA). Antibodies against β-actin and goat anti-rabbit IgG-FITC antibody were supplied by Huaan Biotechnology (Hangzhou, China). FastPure Cell/Tissue Total RNA Isolation Kit, HiScript^®^ III All-in-one RT SuperMix Perfect for qPCR, ChamQ Universal SYBR qPCR Master Mix, and Annexin V-FITC/PI Apoptosis Detection Kit were purchased from Vazyme (Nanjing, China). The reporter plasmids SuperTOPFlash, SuperFOPFlash, pRL-TK, and Dual-Lumi™ II Luciferase Reporter Gene Assay Kit were obtained from Beyotime Biotechnology (Shanghai, China). Other chemical reagents were purchased from Guangzhou chemical reagent factory (Guangzhou, China).

### 4.2. Fungal Material

The endophytic fungus *Phomopsis asparagi* DHS-48 was isolated from fresh root of the mangrove plant *Rhizophora mangle* collected in October 2015 at the Dong Zhai Gang Mangrove Garden on Hainan Island, China. The fungus (strain no. DHS-8) was identified as *Phomopsis asparagi* by a molecular biological protocol via the DNA amplification and sequencing of the ITS region (GenBank Accession no. MT126606). A voucher specimen was deposited at one of the authors’ laboratories (J.X.).

### 4.3. Fermentation, Extraction, and Isolation

The fungi were grown on potato dextrose agar (PDA) by supplementing it with 50 μM sodium butyrate and incubating at a temperature of 28 °C for a duration of 7 days. Next, a solitary colony was introduced into sterilized rice solid-substrate medium in Erlenmeyer flasks (130 × 1 L), with each flask comprising 100 g of rice, 100 mL of 0.3% saline water, and 50 μM sodium butyrate. The mixture was then fermented at a temperature of 28 °C for a duration of 28 days. A total of 130 flasks containing cultures were subjected to three rounds of extraction using 400 mL of ethyl acetate (EtOAc) each time. The resulting filtrate was then evaporated under reduced pressure, resulting in a crude extract weighing 20 g. The EtOAc extracts were chromatographed by silica gel column chromatography (CC) using a step gradient elution with CH_2_Cl_2_–MeOH (30:1, 25:1, 20:1, 15:1, 10:1, 8:1, 6:1, 3:1, 1:1) to provide nine fractions (Fr. 1–Fr. 9). Fr. 3 was subjected to open silica gel CC using gradient elution with CH_2_Cl_2_–EtOAc (6:1, 5:1, 4:1, 3:1, 2:1, 1:1, 1:2, *v*/*v*) to yield seven fractions (Fr. 3.1–Fr. 3.7). Fr. 3.2 was subjected to open silica gel CC using gradient elution with CH_2_Cl_2_–EtOAc (3:1, *v*/*v*) to yield DD-9 (50 mg).

### 4.4. Cell Culture

HepG2 and HeLa cell lines were purchased from the Type Culture Collection of the Chinese Academy of Sciences (Shanghai, China). The rat liver BRL-3A cell line was purchased from Shanghai Gaining Biological Technology Co., Ltd. (Shanghai, China). HEK-293T (human kidney cell line) was from Beyotime Biotechnology (Shanghai, China). HepG2, HeLa, BRL-3A, and HEK-293T were all cultured in MEM medium with 10% FBS and 1% penicillin and streptomycin mixture. Cells were maintained in a humidified atmosphere with 5% CO_2_ at 37 °C.

### 4.5. MTT Assay

Cell viability was detected by the MTT method. Briefly, HepG2, HeLa, and BRL-3A cells in the logarithmic growth phase were digested with trypsin and seeded in 96-well plates at a density of 6 × 10^3^ cells per well and underwent 24 h/48 h DD-9 treatments, and were then incubated in 0.5 mg/mL MTT for 4 h at 37 °C. Afterward, the supernatant was discarded, and 200 μL DMSO was added to dissolve the MTT formazan crystals. After shaking gently on the shaker, the absorbance was measured at a wavelength of 570 nm.

### 4.6. Cell Migration Assay

HepG2 and HeLa cells were seeded in a 6-well plate at 5 × 10^5^ cells per well to confluent monolayers, which then were starved in serum-free medium overnight. Straight wounds were made by using 200 µL pipette tips. After washing with medium to remove cell debris, 5 μM DD-9 or 0.1% DMSO was added and migration of the cell monolayer from the edge of the scratch was photographed at regular intervals (0, 12, 24, and 48 h) and analyzed using ImageJ v1.54j software.

### 4.7. Transwell Assays

Cells (2 × 10^5^ cells per well) were suspended in 200 µL serum-free medium and seeded into the upper chamber in the absence and presence of DD-9 (5 μM), and 600 µL medium containing 20% fetal bovine serum was added at the bottom chamber as a chemoattractant. Cells were incubated in a 5% CO_2_ and 37 °C incubator for 24 h. The upper non-invading cells were removed with cotton swabs. The cells were fixed in methanol for 10 min and stained with 0.1% crystal violet for 20 min, and then photographed microscopically. Bound crystal violet was dissolved with 33% acetic acid and absorbance was measured at 570 nm for quantitative analysis. For invasion assays, the chambers with 8 μM-pore membranes were coated with 20 μL Matrigel. The remaining procedures were identical to those of the migration assay.

### 4.8. Colony Formation Assay

Cells were seeded in a 6-well culture plate at a density of 3 × 10^4^ cells per well and treated with various concentrations of DD-9 (2, 4, and 8 μM) or 0.1% DMSO. After the plates were cultured at 37 °C for 14 days, cells were fixed in 4% paraformaldehyde and stained with 0.1% crystal violet. Stained colonies consisting of more than 50 cells were counted under a dissecting microscope to determine the clonal formation efficiency.

### 4.9. Apoptosis Assay

To determine the apoptosis caused by DD-9 in HepG2 and HeLa cells, Annexin V-FITC/PI double staining assay was performed. Briefly, cells (2 × 10^5^ cells per well) were seeded in a 24-well plate and subsequently treated with DD-9 (2, 4, and 8 μM) or 0.1% DMSO at 37 °C for 48 h, after which the cells were washed with PBS and trypsinized. Next, the staining dye of Annexin V (5 μL) and PI (5 μL) was added to cells and incubated in the dark for 15 min. Finally, the stained cells were analyzed using flow cytometer (excitation/emission wavelengths 488/535 nm).

### 4.10. Cell Cycle Assay

The cell cycle distribution in the presence of DD-9 was measured using flow cytometry. Cells (2 × 10^5^ cells per well) were seeded in a 6-well plate and incubated in the presence of DD-9 (4, 8 μM) or 0.1% DMSO at 37 °C for 48 h. After that, the adherent cells were washed with PBS, harvested with trypsin, collected by centrifugation, and rewashed via PBS. Then, the cells were stained with DNA staining solution (1 mL) and permeabilization solution (10 μL), and then incubated at room temperature in the dark for 30 min. Finally, the cell-cycle distribution was analyzed by flow cytometry at 488 nm excitation using FlowJo^TM^ v10.8.1 software.

### 4.11. Luciferase Reporter Gene Assay

For TOP/FOPFlash luciferase reporter assay detecting the activity of the Wnt/β-catenin signaling pathway, the HEK293 reporter (TOPFlash) cells were inoculated into 24-well plates at a density of 5 × 10^5^ cells per well and transfected with SuperTopFlash or SuperFopFlash (0.25 μg) and internal control plasmid pRL-TK (2.5 μg) using Lipo293™. After transfection for 24 h, DD-9 (2, 4, 8 μM) or 0.1% DMSO were added and incubated for 24 h. Next, the cells were lysed and collected for firefly luciferase activity assay using the Dual-Lumi^TM^ II Luciferase Reporter Gene Assay Kit according to the procedure provided by the manufacturer.

### 4.12. qPCR Analysis

After treatment with 5 μM DD-9 or 0.1% DMSO for 24 h, the total RNAs were isolated from HepG2 and HeLa cells using the FastPure Cell/Tissue Total RNA Isolation Kit and then reverse-transcribed with the HiScript III All-in-one RT SuperMix Perfect for qPCR supplied by Vazyme Co., Ltd. (Nanjing, China). The resulting cDNAs were amplified on the ABI StepOne Plus Real-time Detection System (Thermo Fisher, Waltham, MA, USA) with SYBR Green qPCR Master Mix (High ROX), and Glyceraldehyde-3-phosphate dehydrogenase (GAPDH) was used as the internal control. Primers were:

β-catenin: forward 5′-CATCTACACAGTTTGATGCTGCT-3′ and reverse 5′-GCAGTTTTGTCAGTTCAGGGA-3′.

CyclinD1: forward 5′-AATGACCCCGCACGATTTC-3′ and reverse 5′-TCAGGTTCAGGCCTTGCAC-3′.

LEF1: forward 5′-AGGAACATCCCCACACTGAC-3′ and reverse 5′-AGGTCTTTTTGGCTCCTGCT-3′.

GAPDH: forward 5′-CCAGAACATCATCCCTGCCTCTACT-3′ and reverse 5′-GGTTTTTCTAGACGGCAGGTCAGGT-3′.

### 4.13. Immunocytochemical Staining

HepG2 and HeLa cells were treated with 5 μM DD-9 or 0.1% DMSO for 48 h, fixed with 4% PFA for 30 min, and then subjected to osmotic treatment with 0.1% Triton X-100 for 15 min. The cells were blocked with 5% BSA for 1 h and then incubated with primary antibody β-catenin overnight. After incubation, the cells were washed three times and incubated with fluorescent secondary antibody at room temperature for 1 h. After washing, the cells were stained with DAPI in a dark room, and the stained cells were observed by laser confocal microscopy.

### 4.14. Western Blot Analysis

After treatment with DD-9 (2, 4, 6, 8 μM) or 0.1% DMSO for 24 h, cells were collected and lysed in a RIPA buffer (Boster, Wuhan, China) containing PMSF, followed by centrifugation at 10,000× *g* for 10 min at 4 °C. The resulting supernatant was collected and protein concentration was quantified via BCA Protein Assay Kit (Boster, Wuhan, China). Nuclear proteins were extracted using the Cell Structure Nuclear and Cytoplasmic Protein Extraction kit (Beyotime, Shanghai, China), according to the manufacturer’s protocol. SDS-PAGE was carried out for the separation of proteins, followed by electroblotting onto the PVDF (polyvinylidene fluoride) membrane. The membrane was blocked with TBST containing 5% skimmed milk powder at room temperature for 1 h, and then incubated overnight with the primary antibodies recognized as GSK3-β, p-GSK3-β, β-catenin, β-actin, cyclin D1, and c-Myc at 4 °C. Secondary anti-rabbit IgG and HRP-linked antibody (Cell Signaling Technology, Danvers, MA, USA) were then employed to probe the blots for 1 h at room temperature. Finally, an enhanced chemiluminescence (ECL) reagent (Boster, Wuhan, China) was used to visualize immunoreactivity.

### 4.15. Molecular Docking Studies

The AUTODOCK 4.2 software was employed for conducting molecular docking experiments. The compound DD-9, sourced from PubChem (www.ncbi.nlm.nih.gov/pccompound), accessed on 6 April 2024, underwent Gaussian09 minimization to ensure stability and facilitate the docking process. The X-ray crystal structures of β-catenin (PDB ID: 1JDH, 1.90 Å) and GSK3-β (PDB ID: 4NM7, 2.30 Å) proteins were retrieved from the Protein Data Bank (PDB) (www.rcsb.org), accessed on 6 April 2024. Water molecules were removed through GROMACS minimization 4.5 prior to docking analysis. The resulting postures from the docking simulations were visualized and analyzed using Discovery Studio Visualizer 3.5.

### 4.16. Statistical Analysis

IBM SPSS Statistics 29.0 software was used for independent sample analysis and *p* value calculation. GraphPad Prism 5.0 software was used for statistical analysis of the results. Statistics were presented as mean ± standard deviation, with * *p* < 0.05 considered significant, and ** *p* < 0.01 considered extremely significant.

## 5. Conclusions

Taken together, DD-9, a bioactive compound obtained from chemical epigenetic manipulation of mangrove endophytic fungus *Phomopsis asparagi* DHS-48, may suppress proliferation and induce apoptosis of liver and cervical cancer cells, possibly at least in part via GSK-3β-mediated crosstalk with the Wnt/β-catenin signaling axis, providing insights into the mechanism for the potency of DD-9 on HCC and CC and laying a theoretical basis for this agent as a potential antitumor product.

## Supplementary Materials

The following supporting information can be downloaded at: https://www.mdpi.com/article/10.3390/md22060278/s1, Figure S1: Protein Molecular Weight Marker used in western blotting system to detect c-Myc(60kda), cyclin D1(36kda), GSK3-β(46kda), p-GSK3-β(46kda), β-catenin(92kda), β-actin(45kda); Figure S2: Protein Molecular Weight Marker used in western blotting system to detect nucl-β-catenin (92kda), histone (17kda) uses maker; Figure S3: Original protein image of c-Myc (60kda) detected using the Amersham imager 600 QC; Figure S4: Original protein image of cyclin D1 (36kda) detected using the Amersham imager 600 QC; Figure S5: Original protein image of GSK3-β (46kda) detected using the Amersham imager 600 QC; Figure S6: Original protein image of p-GSK3-β (46kda) detected using the Amersham imager 600 QC; Figure S7: Original protein image of β-catenin (92kda) in HepG2 cells detected using the Amersham imager 600 QC; Figure S8: Original protein image of β-catenin (92kda) in Hela cells detected using the Amersham imager 600 QC; Figure S9: Original protein image of nucl-β-catenin (92kda) in HepG2 cells detected using Tanon 5200; Figure S10: Original protein image of nucl-β-catenin (92kda) in Hela cells detected using Tanon 5200; Figure S11: Original protein image of β-actin (45kda) detected using the Amersham imager 600 QC; Figure S12: Original protein image of Histone (17kda) in HepG2 cells detected using Tanon 5200; Figure S13: Original protein image of Histone (17kda) in Hela cells detected using Tanon 5200.
