# Supplementary material for: Dicerandrol C Suppresses Proliferation and Induces Apoptosis of HepG2 and Hela Cancer Cells by Inhibiting Wnt/β-Catenin Signaling Pathway
Source: Mar Drugs. 2024 Jun 14;22(6):278. doi: 10.3390/md22060278 (PMC11204528; doi:10.3390/md22060278)
Supplement: Supplementary file 1 [file marinedrugs-22-00278-s001.zip › marinedrugs-3049793-supplementary.pdf]

Supplementary Material:

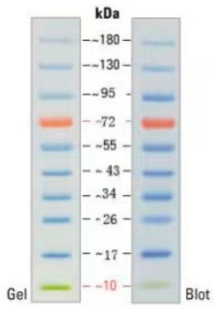

Figure S1. Protein Molecular Weight Marker used in western blotting system to detect c-Myc(60kda), cyclin D1(36kda), GSK3-β(46kda), p-GSK3-β(46kda), β-catenin(92kda), β-actin(45kda)

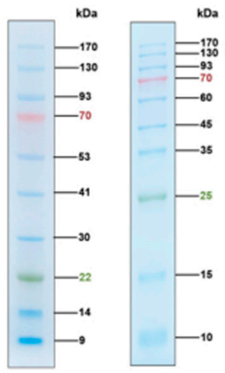

Figure S2. Protein Molecular Weight Marker used in western blotting system to detect nucl-β-catenin (92kda) , histone (17kda) uses maker

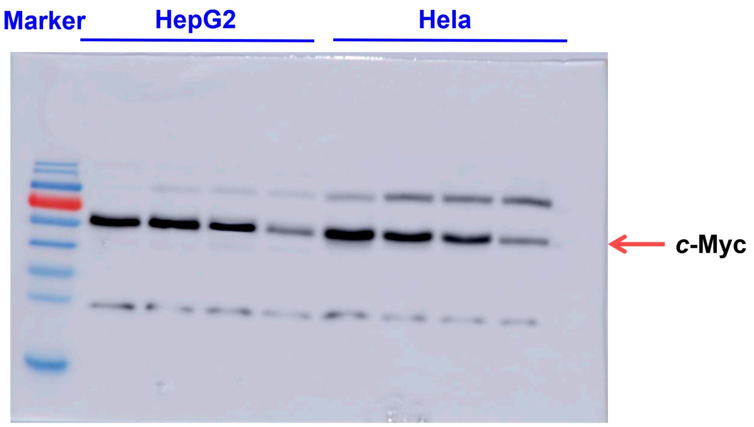

Figure S3. Original protein image of c-Myc (60kda) detected using the Amersham imager 600 QC

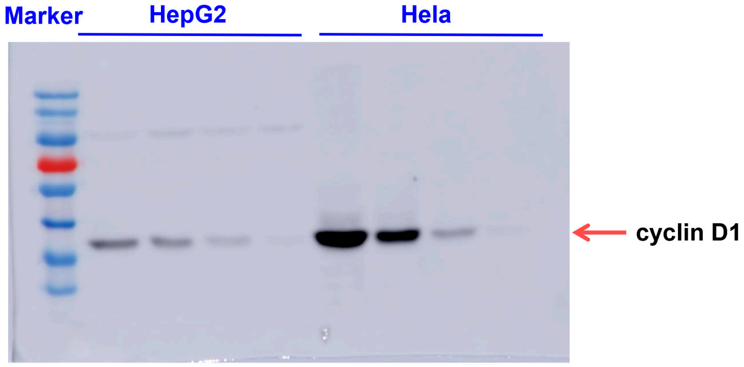

Figure S4. Original protein image of cyclin D1 (36kda) detected using the Amersham imager 600 QC

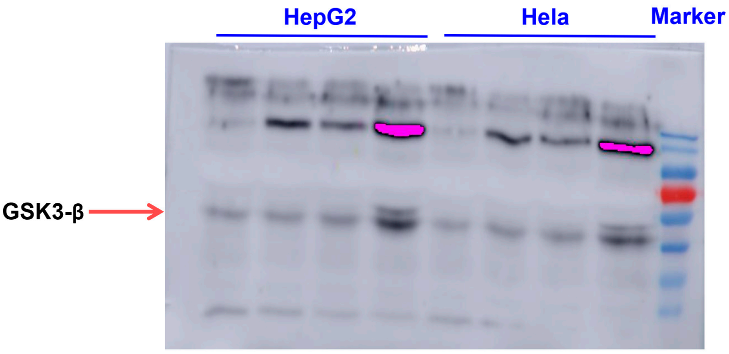

Figure S5. Original protein image of GSK3-β (46kda) detected using the Amersham imager 600 QC

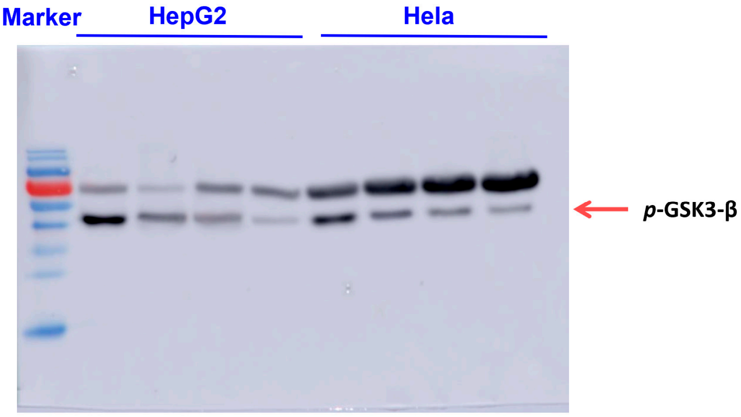

Figure S6. Original protein image of *p*-GSK3-β (46kda) detected using the Amersham imager 600 QC

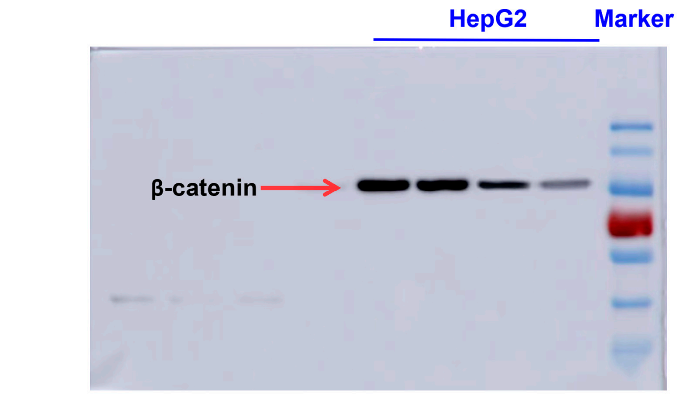

Figure S7. Original protein image of β-catenin (92kda) in HepG2 cells detected using the Amersham imager 600 QC

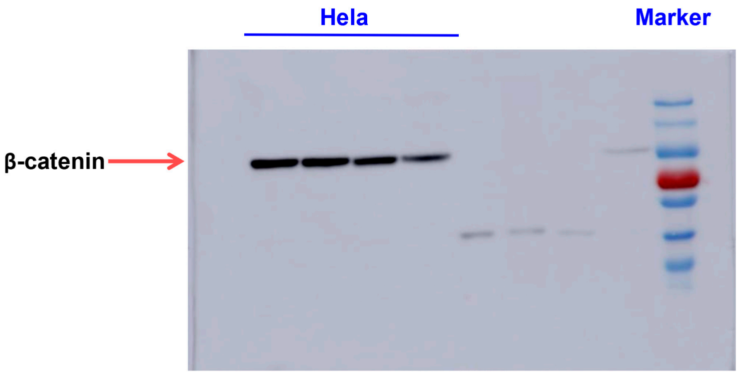

Figure S8. Original protein image of β-catenin (92kda) in Hela cells detected using the Amersham imager 600 QC

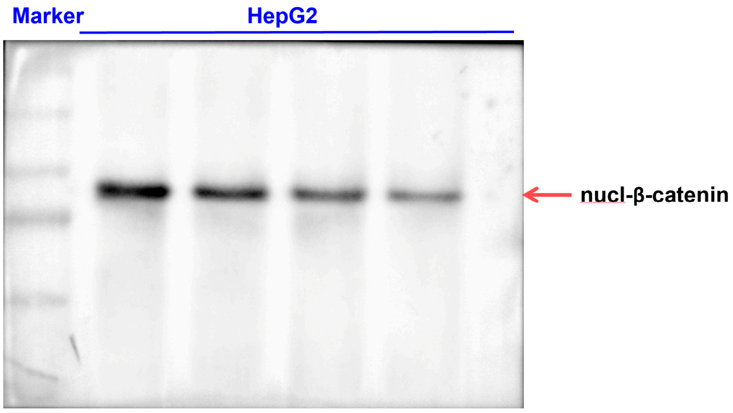

Figure S9. Original protein image of nucl-β-catenin (92kda) in HepG2 cells detected using Tanon 5200

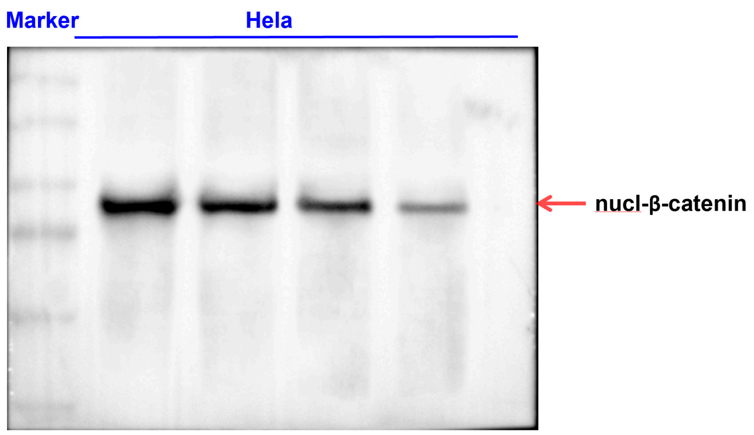

Figure S 10. Original protein image of nucl-β-catenin (92kda) in Hela cells detected using Tanon 5200

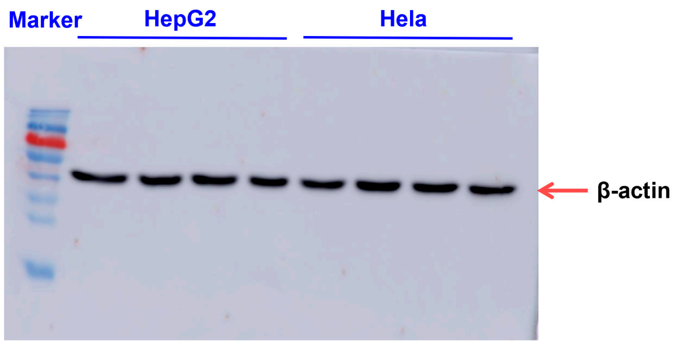

Figure S11. Original protein image of β-actin (45kda) detected using the Amersham imager 600 QC

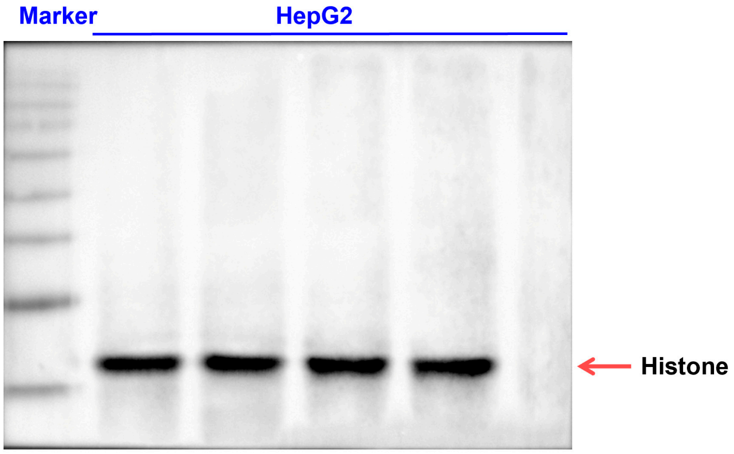

Figure S12. Original protein image of Histone (17kda) in HepG2 cells detected using Tanon 5200

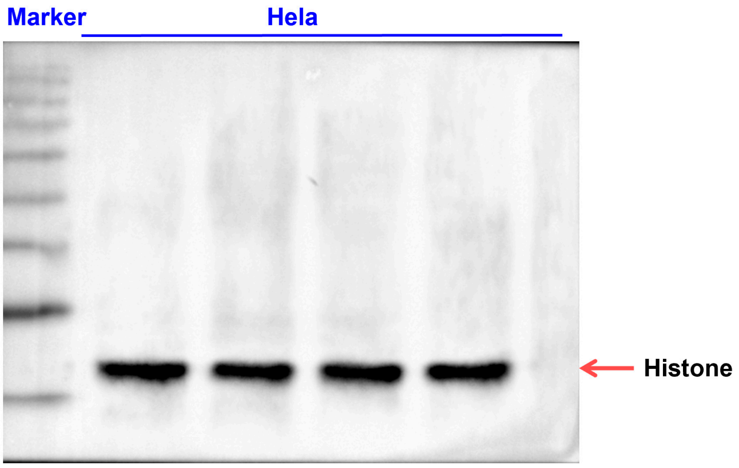

Figure S13. Original protein image of Histone (17kda) in Hela cells detected using Tanon 5200
